# Hydrodissection Facilitates Open Resection of Morton’s Neuroma Through a Plantar Approach: Technique Tip

**DOI:** 10.1177/24730114241274778

**Published:** 2024-08-31

**Authors:** Sufyan Faridi, Amanda Vandewint, Jacob Matz

**Affiliations:** 1 Faculty of Medicine, Dalhousie Medicine New Brunswick, Saint John, NB, Canada; 2 Canada East Foot & Ankle, Saint John, NB, Canada; 3 Division of Orthopaedic Surgery, Department of Surgery, Dalhousie University/DMNB, Saint John, NB, Canada; 4 Horizon Health Network, Saint John, NB, Canada

**Keywords:** Morton’s neuroma, hydrodissection, neurectomy, plantar approach

## Abstract

**Visual Abstract:**
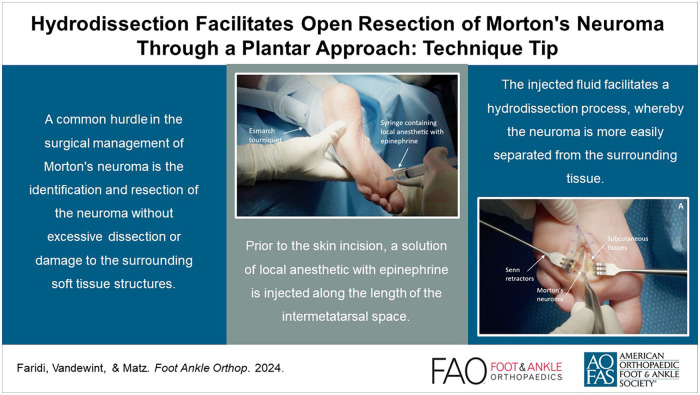
This is a visual representation of the abstract.

This is a visual representation of the abstract.

## Introduction

Morton’s neuroma is a condition characterized by benign thickening of the plantar digital nerves.^5^ This perineural fibrosis, primarily affecting the plantar digital nerves in the second and third intermetatarsal spaces, leads to neuropathic symptoms of radiating or burning pain, numbness in the toes, and a sensation of walking on a pebble.^4^

A common hurdle in the surgical management of Morton’s neuroma is the identification and resection of the neuroma without excessive dissection or damage to the surrounding soft tissue structures.^1^ In the quest for enhanced surgical outcomes, we propose hydrodissection as a promising adjunct technique. Hydrodissection involves the injection of fluid around the neuroma, creating a plane of separation between the neuroma and surrounding tissues.^3^ This technique has the potential to facilitate the subsequent surgical resection by enhancing the visualization and identification of the neuroma, thereby minimizing collateral tissue damage.

This technique tip describes the application of hydrodissection in the surgical management of Morton’s neuroma through a plantar approach, thus representing a novel modified approach to the treatment of this challenging condition.

## Technique

Anesthesia for this procedure consists of either general anesthetic or awake surgery with a regional ankle block. The patient is secured in a semi-prone position with prepping and draping of the leg undertaken in a usual sterile fashion (Supplemental Video 1).^7^ An Esmarch ankle tourniquet is applied generating a pressure of approximately 300 mm Hg to achieve ischemia.

**Figure 1.**
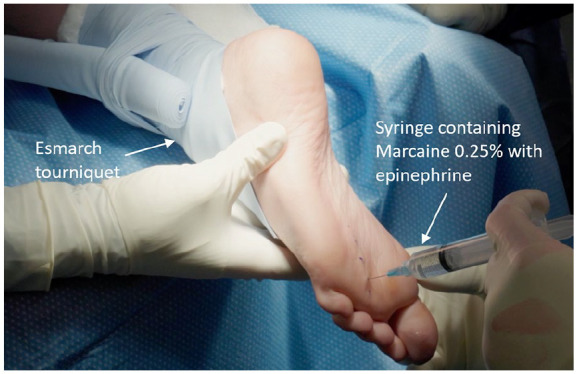
Hydrodissection step involving injection of 10 mL of Marcaine 0.25% with epinephrine solution (1:200 000) into the intermetatarsal space using a 25-gauge needle.

The two metatarsal heads between which the neuroma is located (ie, the second and third heads, or the third and fourth heads) are palpated, and a 3-4-cm-long, longitudinal incision is marked in the intermetatarsal space on the plantar aspect. A 10-mL syringe containing the hydrodissection solution of Marcaine 0.25% with epinephrine (1:200 000) is used to simultaneously confirm the placement of the mapped incision by palpating against the metatarsal shafts with the needle, while injecting the solution along the length of the intermetatarsal space (Figure 1). The injection begins at the level of the metatarsal cortex and is continued as the needle is withdrawn to the subcutaneous level. The injected fluid facilitates a hydrodissection process, whereby the nerve tissue will be more easily separated from the surrounding tissue. The epinephrine component of the solution provides the additional benefit of delivering improved hemostasis.

**Figure 2.**
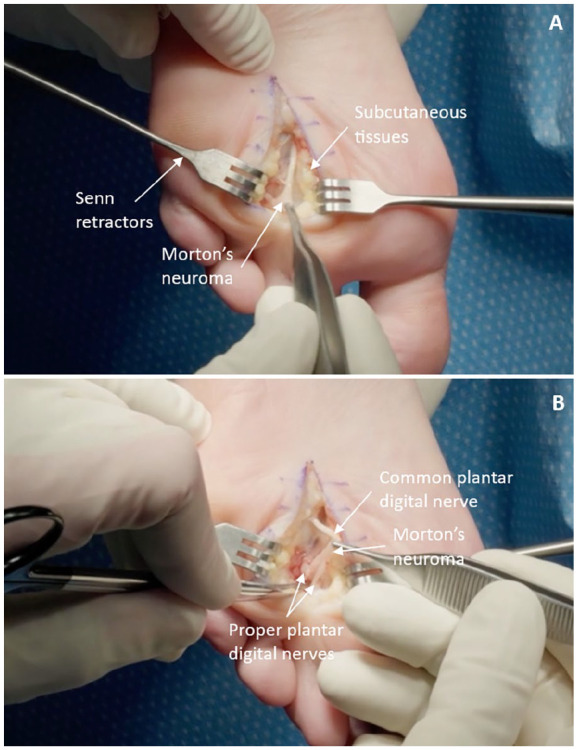
(A) Visualization of the common plantar digital nerve with the Morton’s neuroma positioned at the distal end. (B) View of the bifurcation of the common plantar digital nerve distal to the site of the Morton’s neuroma into the proper plantar digital nerve branches.

Following dissection of the skin and subcutaneous tissue, the nerve including the neuroma is exposed when separated out from the surrounding tissues, a step noticeably facilitated by the preceding hydrodissection component of the procedure (Figure 2). After excising the neuroma, the fatty tissue is reapproximated with 3-0 Monocryl followed by subcutaneous closure with 3-0 Monocryl. The skin is then evenly aligned with 3-0 Nylon sutures passed in a vertical mattress configuration. A soft dressing is applied and the operative foot is kept heel weightbearing for 3 weeks in a postoperative shoe followed by transition to weightbearing as tolerated in a regular shoe.^1,6^

## Discussion

We propose in this technique tip that hydrodissection facilitates a more precise and efficient neuroma resection by creating a clear demarcation between the nerve tissue and the surrounding soft tissue structures. This precision is paramount, as it facilitates a more complete resection while minimizing the risk of collateral damage to surrounding tissues, potentially leading to reduced postoperative pain, faster recovery times, and decreased likelihood of recurrence.^1,2^

Complications associated with the plantar approach tend to be wound healing delays, hypertrophic scar formation, and paresthesias.^1^ The technique illustrated carries the potential of optimizing outcomes in several ways. First, by landmarking the incision during the injection process, the placement of the incision is optimized, avoiding areas of high pressure beneath the metatarsal heads. Second, by exploiting the potential space around the plantar nerve, hydrodissection has the potential of making the procedure more efficient, requiring less invasive dissection, which may help reduce the chances of wound healing complications. Finally, by making the identification of the neuroma simpler, the chances of an inadequate resection are reduced.

Early experiences in 6 patients using this hydrodissection technique have demonstrated its inclusion as beneficial. In comparison to the traditional technique previously used by the senior author, we find that hydrodissection allows for more efficient and less traumatic neuroma excision, with favorable postoperative outcomes. Although this technique demonstrates promising outcomes, it is imperative to consider the need for further research with larger cohorts and longer follow-up periods. These investigations will allow for better elucidation of the long-term benefits and possible complications associated with hydrodissection in the resection of Morton’s neuroma.

In conclusion, hydrodissection provides a simple novel adjunct to the surgical management of Morton’s neuroma that offers the potential of making neuroma identification and resection more efficient and reliable, while decreasing morbidity to adjacent structures.

## Supplemental Material

sj-pdf-1-fao-10.1177_24730114241274778 – Supplemental material for Hydrodissection Facilitates Open Resection of Morton’s Neuroma Through a Plantar Approach: Technique Tip Supplemental material, sj-pdf-1-fao-10.1177_24730114241274778 for Hydrodissection Facilitates Open Resection of Morton’s Neuroma Through a Plantar Approach: Technique Tip by Sufyan Faridi, Amanda Vandewint and Jacob Matz in Foot & Ankle Orthopaedics
